# Initial Field Validation of Poroelastic Pavement Made with Crumb Rubber, Mineral Aggregate and Highly Polymer-Modified Bitumen

**DOI:** 10.3390/ma13061339

**Published:** 2020-03-15

**Authors:** Piotr Jaskula, Jerzy Ejsmont, Marcin Stienss, Grzegorz Ronowski, Cezary Szydlowski, Beata Swieczko-Zurek, Dawid Rys

**Affiliations:** 1 Faculty of Civil and Environmental Engineering, Gdansk University of Technology, 80-233 Gdańsk, Poland; pjask@pg.edu.pl (P.J.); marcin.stienss@pg.edu.pl (M.S.); cezary.szydlowski@pg.edu.pl (C.S.); dawrys@pg.edu.pl (D.R.); 2 Mechanical Faculty, Gdansk University of Technology, 80-233 Gdańsk, Poland; gronowsk@pg.edu.pl (G.R.); beazurek@pg.edu.pl (B.S.-Z.)

**Keywords:** road, tire, poroelastic pavement, highly modified bitumen, rubber, tire/road noise, rolling resistance, fire

## Abstract

Tire/road noise in most driving conditions dominates other sources of traffic noise. One of the most efficient ways of reducing tire/road noise is to use the so-called “low noise pavement”. According to numerous studies, at present, poroelastic road pavement that is composed of rubber and mineral aggregate and polyurethane or bituminous binder gives the best noise reduction up to 12 dB. Unfortunately, there are many problems with making durable poroelastic pavements. This article presents the first results of a project that is executed in Poland and aims at the development of a durable, low noise poroelastic pavement based on polymer-modified asphalt binder called Safe, Eco-friendly POroelastic Road Surface (SEPOR). Two test sections were built in 2019 to test the production technology and performance of the SEPOR pavement. It is observed that some of the problems with previous poroelastic materials were mainly eliminated (especially delamination from the base layer and raveling) but noise reduction is a little less than expected (up to 9 dB). Rolling resistance for car tires is acceptable and fire properties (damping of spill fuel fires, toxic gas emission) are very good.

## 1. Introduction

Tire/road noise in most driving conditions dominates other sources of traffic noise. The best method to lower tire/road noise is to use road pavements that reduce the efficiency of noise generation mechanisms related to airflow in tire tread grooves and impacts of tire tread elements interfering with the pavement. A typical way of reducing airflow-related mechanisms is to provide good ventilation of tread channels by the porous structure of the pavement and “open” grooves layout. Impact-related mechanisms may be reduced by using smaller aggregate in pavement mixtures as well as by lowering pavement stiffness. As it results from the above, to reduce tire/road noise it is beneficial for the pavement to have a porous structure and at the same time be flexible. Poroelastic Road Surfaces (PERS), which are currently in the experimental stage, are characterized by such features.

According to [1], a Poroelastic Road Surface is a wearing course for roads with a very high content of interconnecting voids so as to facilitate the passage of air and water through it, while at the same time, the surface is elastic due to the use of rubber (or other elastic products) as the main aggregate. The design air void content is at least 20% by volume and the design rubber content is at least 20% by weight.

Generally, there are two different variants of PERS. The first one contains only rubber aggregate (in the form of granules or fibers) and elastic binder (bitumen or polyurethane type). The second variant that seems to be more promising due to higher skid resistance contains also mineral aggregate. In both cases, the most important attributes of the final PERS material are its high elasticity and open voids making the surface porous.

So far, some of the tested PERS pavements have shown unrivaled performance in terms of noise reduction for car tires [2,3]. Compared to reference surfaces, noise reduction is up to 12 dB, making the PERS surface the quietest pavement that exists. The rolling resistance of passenger car tires on PERS surfaces is similar to the existing asphalt and concrete pavements but unfortunately on the high side of it. Regrettably, the rolling resistance of truck tires is significantly higher on PERS surfaces than on classic surfaces. An additional valuable feature of PERS pavements is its positive impact on safety in the event of a vehicle fire associated with spillage of fuel [4]. A PERS surface prevents the spread of fire and reduces its intensity (size of the flames) and even leads to smaller smoke contribution [5]. This feature promotes the use of these surfaces in tunnels and petrol stations. The results obtained in Sweden indicate that PERS also has favorable friction properties in winter, limiting the danger of black ice. Another important and very positive aspect of poroelastic road pavements is their contribution to scrap rubber recycling.

Up to now, the basic problem with PERS is the relatively low durability of this pavement [2]. This problem manifests itself both by debonding of the PERS surface from its base layer and by degrading of the PERS material by the loosening of the aggregate (raveling).

So far, most of the trials with PERS were performed with polyurethane as a mixture binder. This type of binder is relatively difficult to use in road conditions as its curing process is very sensitive to temperature and humidity. What is more, it is not easy to provide high adhesion between the stiff bituminous base layer and elastic polyurethane-based PERS. In the opinion of the authors of this paper, to improve the durability of PERS, some changes in the method of joining this pavement layer to the under-layers should be developed. Improvement may be achieved by introducing an additional reinforced interface layer optimized on one side to bind to the asphalt pavement and on the opposite side to be joined to PERS. It is also important to test the possibility of using a binder different than commonly used polyurethane that will join the mineral aggregate with the rubber aggregate in a better way. It is anticipated that the polymer-modified asphalt binder will help to solve problems related to binding poroelastic material to the rigid base layer.

The sources of raveling of poroelastic mixtures observed in previous studies [6,7] are similar to sources of porous asphalt damage. Raveling is mostly unrelated to pavement structural design, as it primarily depends on surface-contact mechanics and the quality of the skeleton of the mixture [8,9]. Recommendations proposed by [10] suggest that the durability of porous mixtures can be improved by the application of bitumen with high elasticity, including highly modified bitumen. This statement was also confirmed in the research performed by Wu et al. [11], where both Styrene-Butadiene-Styrene (SBS)-modified and epoxy-modified bitumens were used as binders in porous mixtures. In poroelastic mixtures, some additional threats appear in comparison to porous asphalt. They are mainly related to the presence of crumb rubber that is added to the mixture in the dry mixing process. Crumb rubber can cause a process of diffusion of light components of bitumen into rubber [12,13]. The resultant bituminous coating on crumb rubber particles increases stiffness (complex modulus) as well as the elastic response [12] which directly corresponds to properties of the mixture [14].

Wearing courses made from poroelastic mixtures are more sensitive to delamination from the lower layer than typical asphalt mixtures. The problem arises from the difference in stiffness modulus of the porous mixture and lower layer. According to calculations presented in [15], the decrease of modulus of elasticity in poroelastic wearing courses causes an increase of shear stresses inside the poroelastic layer and on the interlayer surface. Debonding of the poroelastic layer occurs when the internal shear strength of the material or interlayer shear bond strength is insufficient to bear shear stresses [16,17].

The conclusions of numerous research programs carried out since the PERS was invented in the 1970s [2] may be summarized as: “PERS has very favorable noise properties but at the present stage of development it cannot provide satisfactory longevity.” In 2018, the new Polish project—Safe, Eco-friendly POroelastic Road Surface (SEPOR) was started. The project differs from most previous projects in respect of the binder used in the poroelastic mix. Instead of using polyurethane it was decided to use bituminous binders, bitumen modified by SBS or rubber [18], and to build a more elaborate intermediate layer between the poroelastic pavement and rigid base course. This article reports the results of the initial tests performed on the first test sections built within this project. In order to clearly distinguish between poroelastic pavements using polyurethane binder and pavements with polymer-modified bitumen that were developed within the SEPOR project, the latter will be designated as “*SEPOR*” instead of “*PERS*”. During works on SEPOR poroelastic pavements, both issues of resistances of mixtures to raveling and to delamination were undertaken.

## 2. Materials and Methods

**Figure 1:**
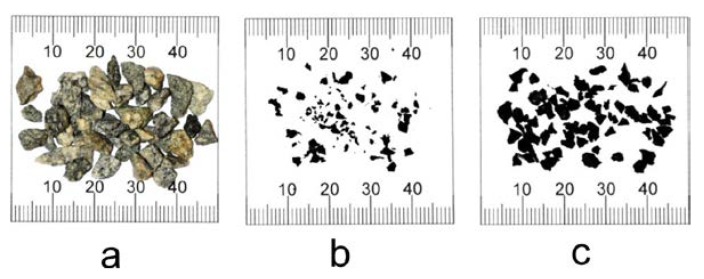
Mineral and crumb rubber materials used in the tests: (**a**) gneiss coarse aggregate 2/5; (**b**) crumb rubber 0.5/2; (**c**) crumb rubber 1/4.

The mineral part of the poroelastic mixture that was investigated contained coarse crushed gneiss aggregate, fine gneiss aggregate and limestone filler. The rubber part of the mixture was composed of crumb rubber obtained from tire recycling. The recycling process used shredding technique at ambient temperature. Different fractions from the standard production of crumb rubber were used in this study: 0.5/2, 1/4 and 4/7 mm. Coarse crushed gneiss aggregate of the 2/5 mm fraction and crumb rubber of different sizes are shown in Figure 1.

Instead of polyurethane epoxy resins used as binders in previous research programs [5,19,20,21] a highly SBS-modified bitumen 45/80-80 containing approximately 7.5% of SBS was used. While conventional modified binder has a continuous bitumen phase, highly modified binder has a continuous polymer phase. SBS-modified bitumen was produced in a refinery and applied in the laboratory and plant mix as a ready-to-use product. The properties of bitumen used during the research program are shown in Table 1.

**Figure 2:**
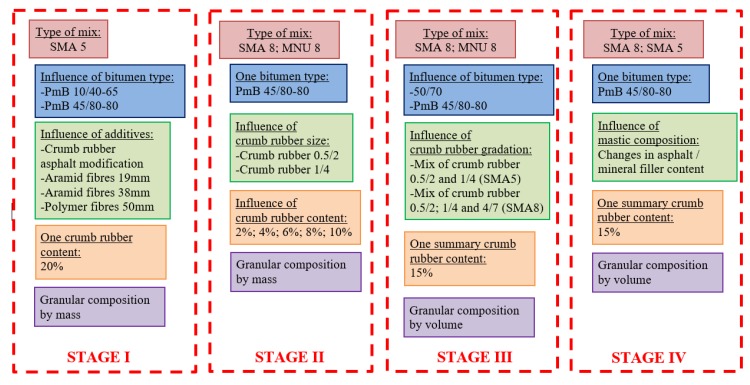
Diagram of poroelastic Safe, Eco-friendly POroelastic Road Surface (SEPOR) mixtures mix design process.

The process of design of adequate poroelastic mixture composition was divided into 4 major stages, reflecting the successive steps of selection and optimization. Every subsequent step was based on the experience gained during the previous stage. A schematic diagram of variables taken into account during each stage is shown in Figure 2. The design of the mineral mixtures was based on existing gradation requirements according to the National Appendix of a standard EN13108 for stone mastic asphalt (SMA) and asphalt concrete for thin wearing course (MNU), some kind of gap-graded asphalt concrete.

During mix optimization, many different variants of poroelastic mixtures were tested. The mineral skeleton of these mixtures was based on traditional asphalt mixtures: stone matrix asphalt, porous asphalt and gap-graded asphalt mixture. The composition of these mixtures was based on Polish and Swedish technical regulations concerning mineral gradation of asphalt mixtures.

At the present stage of the SEPOR project, the authors believe that the total cost of SEPOR will be lower than PERS with a polyurethane binder because of using the highly polymer-modified asphalt binders (HiMA) binder and the possibility to use a typical plant to produce a mixture. Other benefits will be analyzed in the farther stage of the project.

**Figure 3:**
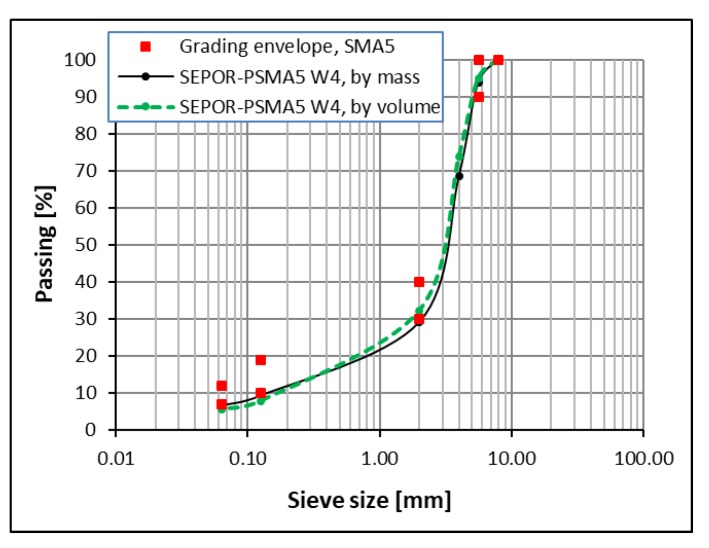
SEPOR-PSMA5 W4 grading curve.

**Figure 4:**
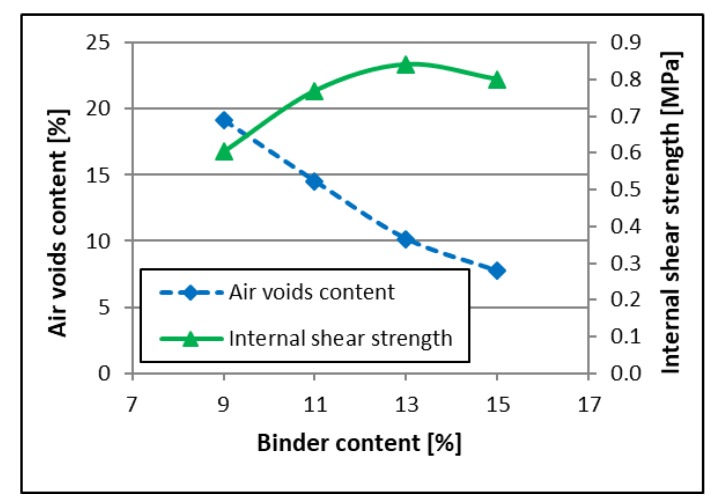
SEPOR-PSMA5 W4 laboratory test results.

As a result, after initial field trials, one poroelastic mixture designated as SEPOR-PSMA5 W4 was selected for trial sections construction. Mixture compositions are presented in Table 2. Figure 3 presents the grading curve and grading envelope. Asphalt–Rubber–Mineral aggregate mixtures with different binder content were tested. After preliminary trials, it was established that two parameters would be taken into consideration in the initial phase of the selection process: (1) air void content and (2) internal (inlayer) shear strength obtained from the Leutner apparatus. It was proposed that the internal shear strength is mainly responsible for the durability of the mixture in the field [22]. The only mixtures that achieved the target levels of these two parameters (air voids content ~15%, internal shear strength ~1 MPa at + 20 °C) were chosen for trial section construction. The test results are presented in Figure 4.

**Figure 5:**
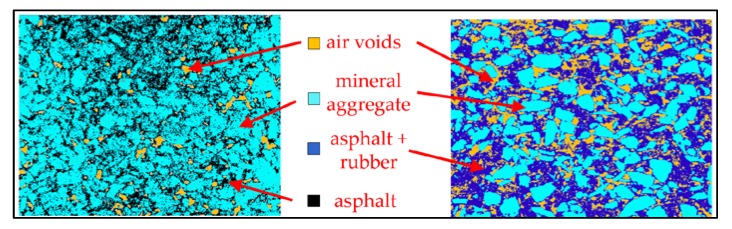
CT scan of the ordinary SMA 5 mixture (**a**) and poroelastic SEPOR-PSMA5 W4 (**b**).

Poroelastic material contains both mineral aggregate and crumb rubber aggregate. To produce this kind of material, not only rubber powder (filler) was used but also chunks with grain from 0.5 mm to 4 mm. Figure 5 presents the CT scan of the ordinary SMA 5 (Stone Mastic Asphalt with a maximum grain size of 5 mm) mixture (a) and poroelastic SEPOR-PSMA5 W4 mixture (b). The crumb rubber aggregate is spread between mineral aggregate in such a way that the contact between mineral coarse aggregate grains is broken lightly enough and, under pressure from vehicles, the load is transferred through the mineral skeleton.

After the laboratory tests phase the poroelastic mixture with 11% of binder content was selected to construct a field test section in Dąbrówka (mixture designation SEPOR-D-PSMA5 W4) and to construct Galaktyczna test section (SEPOR-G-PSMA5 W4, A11). An additional variant with 13% binder was constructed on the Galaktyczna test section (SEPOR-G-PSMA5 W4, A13).

## 3. Construction of Trial Sections

Two trial sections have been constructed. The first one was located on a private internal road in a closed area near Dąbrówka village. Before construction, the existing pavement was demolished to subgrade level. The main purpose of this test section was (1) to evaluate the effectiveness of different poroelastic wearing course bonding techniques to the lower layer, (2) to conduct initial experiments concerning noise reduction and (3) to conduct fire tests concerning fuel spillages. Other aspects of producing and placing poroelastic mixtures were also investigated, such as:

- Adding crumb rubber to the pugmill of the asphalt batch plant by means of belt conveyor Normally used for adding reclaimed asphalt pavement;
- Mixing of the poroelastic mixture in ordinary pugmill of the asphalt plant;
- Using typical asphalt paver for placing a layer of the poroelastic mixture;
- Relation between compaction degree in the field with laboratory compaction results.

The mixing temperature was 180 °C and the initial compaction temperature was 160 °C. The final compaction temperature was 125 °C.

The total length of the Dąbrówka test section was ca. 80 m. The width of the carriageway was 5 m. The poroelastic mixture was laid on both lanes. Because of the adjacent parking lot to which access is provided by the test section, the pavement was subjected not only to load of straight forward moving wheels, but also turning wheels. The total length was divided into five sections, which differed in terms of used wearing course and bonding technique to the lower layer. These were as follows:

- Section 1—Poroelastic SEPOR-D-PSMA5 W4 mixture laid on even (not milled) surface of the binder course, covered with tack coat and glass geogrid reinforcing layer;
- Section 2—Poroelastic mixture laid on longitudinally milled surface of the binder course, with tack coat and glass geogrid reinforcing layer;
- Section 3—Poroelastic mixture laid on longitudinally milled surface of the binder course, with tack coat only (without glass geogrid reinforcing layer);
- Section 4—Poroelastic mixture laid on even (not milled) surface of the binder course, with tack coat only (without glass geogrid reinforcing layer);
- Section 5—Reference section with typical SMA 8 (Stone Mastic Asphalt with a maximum grain size of 8 mm) wearing course laid on even surface of the binder course, with tack coat only.

**Figure 6:**
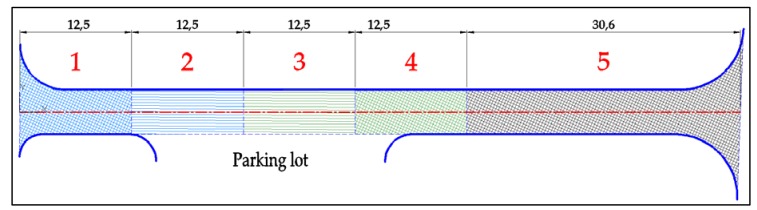
Layout of the Dąbrówka test section. Red numbers designate each section described in text above.

**Figure 7:**
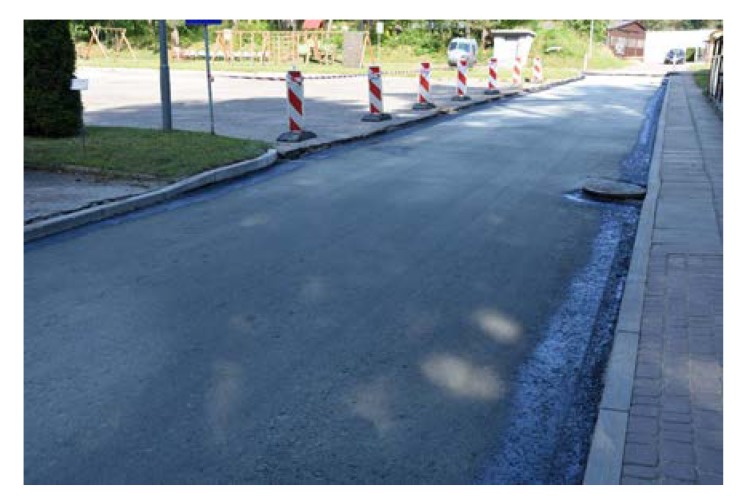
Unbound aggregate base course ready for laying the binder course made of AC 16W.

**Figure 8:**
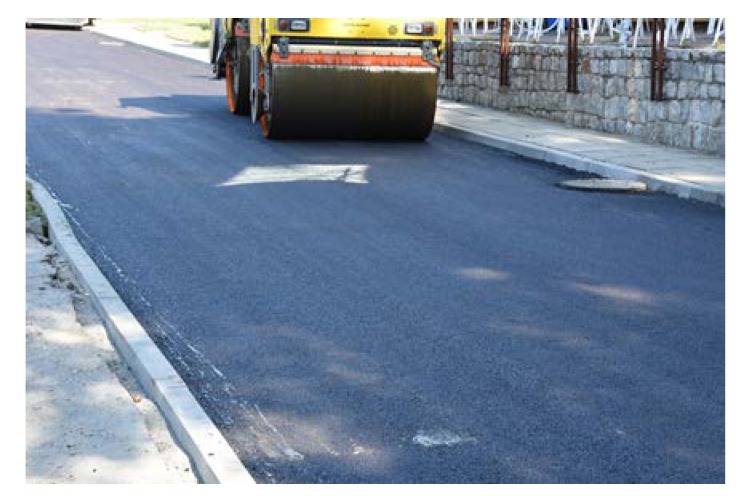
Compaction of the binder course AC 16W.

**Figure 9:**
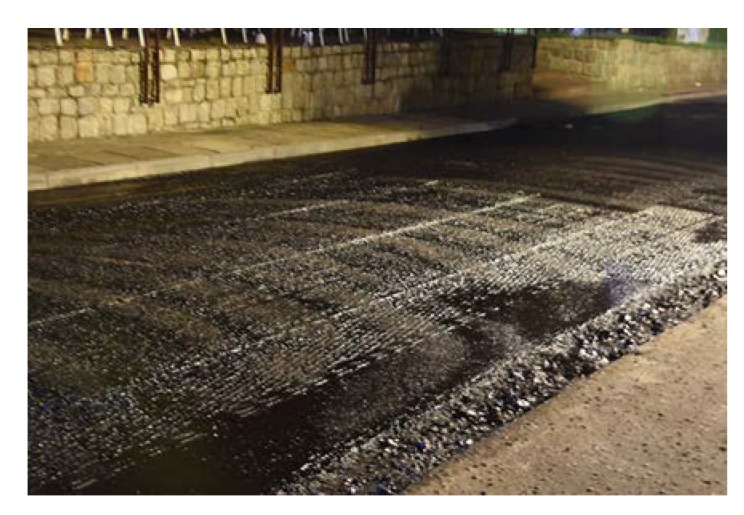
Milled surface of the binder course.

**Figure 10:**
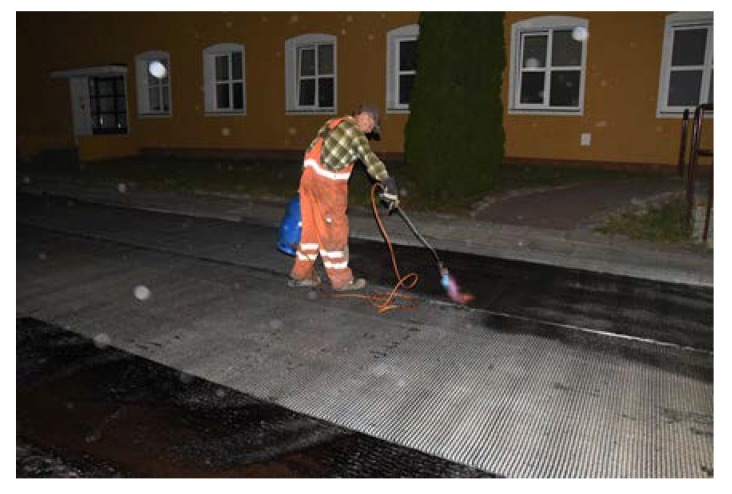
Applying glass geogrid on the surface of the binder course.

**Figure 11:**
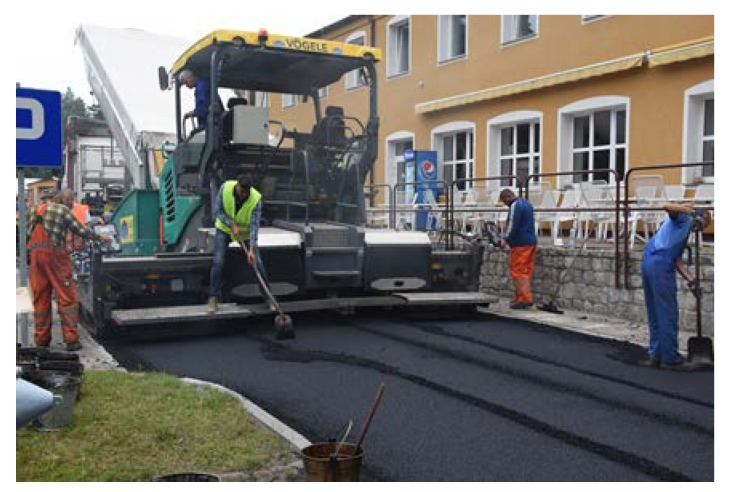
Paving of the poroelastic mixture.

**Figure 12:**
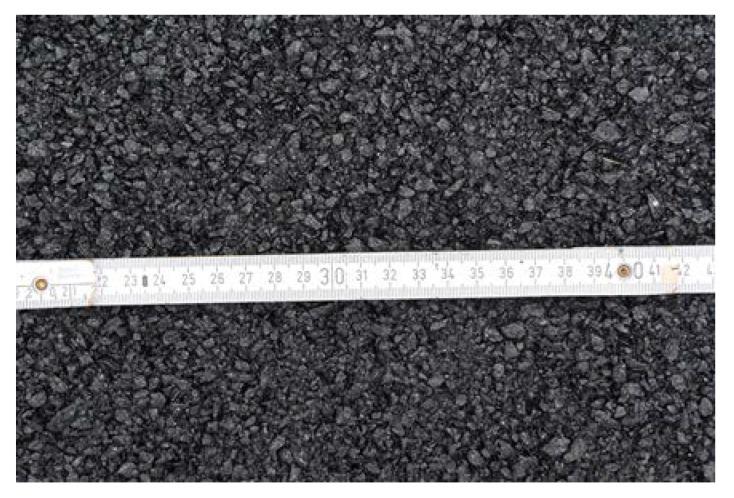
Finished texture of the poroelastic wearing course.

The length of every poroelastic layer section was 12.5 m, the length of the reference SMA section was almost 31 m. For every section, the same type of bitumen emulsion—C 60 BP3 ZM—and amount of bitumen residue—0.2 kg/m^2^—were used. Binder course was made of AC 16W asphalt concrete based on 35/50 neat bitumen. The base course was made of unbound crushed aggregate. The designed thickness of the poroelastic layer was 3.5 cm, binder course 8 cm and base course 20 cm. All asphalt mixtures used for construction (including poroelastic one) were transported from asphalt plan located approximately 50 km from the test site. Transport time did not exceed 60 min. The general layout of the Dąbrówka trial section is shown in Figure 6. Figure 7, Figure 8, Figure 9, Figure 10, Figure 11 and Figure 12 present selected aspects of the construction phase.

Test pads of different types of wearing courses for fire tests were located on the adjacent parking lot. Firstly, the area was covered with an unbound aggregate mixture to achieve a flat and even surface. Afterward, a strip of binder course made of asphalt concrete AC 16W was laid and compacted. On this surface, four rectangular sections of different wearing courses were placed. These included:

- Typical wearing course made of SMA 8 mixture with modified bitumen 45/80-55;
- Porous asphalt wearing course PA 11 with neat bitumen 50/70;
- Poroelastic wearing course made of poroelastic mixture PERS with polyurethane binder;
- Poroelastic wearing course made of designed SEPOR-D-PSMA5 W4 mixture with highly polymer-modified binder HiMA 45/80-80.

**Figure 13:**
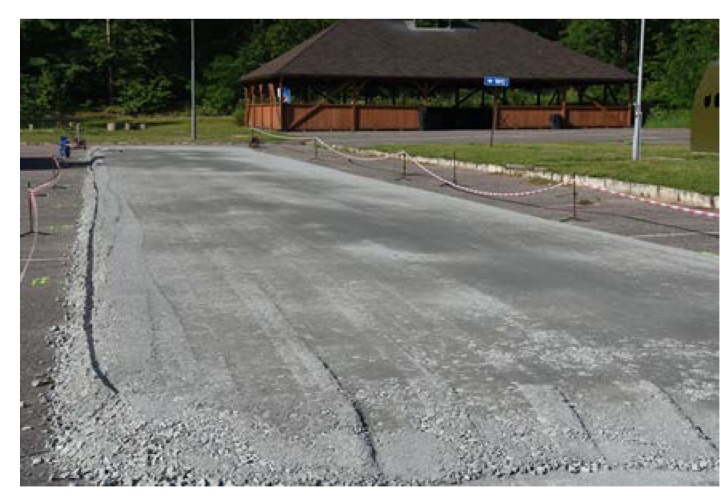
Parking lot area leveled out with the unbound aggregate base course.

**Figure 14:**
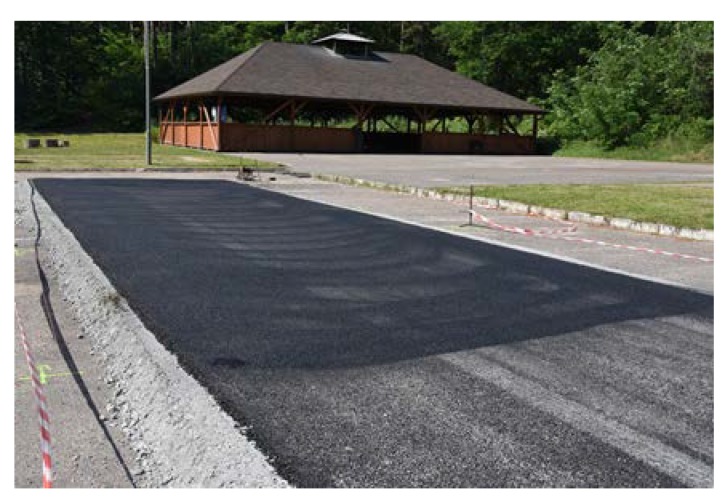
Binder course AC 16W before installation of different types of wearing courses.

**Figure 15:**
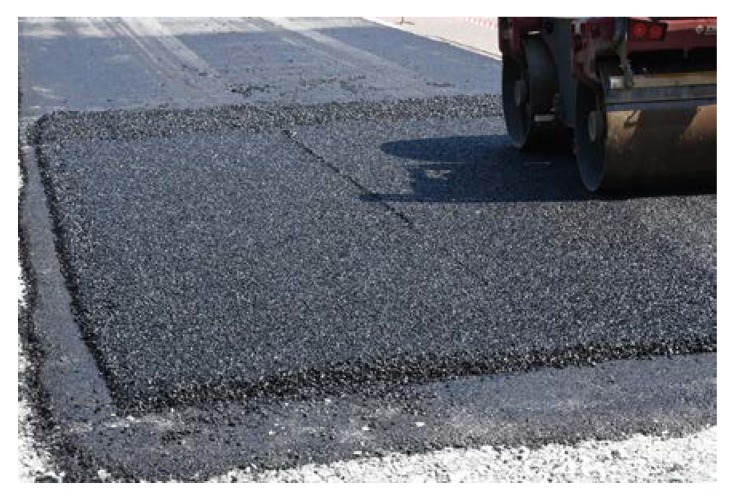
Compacting of PA 11 mixture.

**Figure 16:**
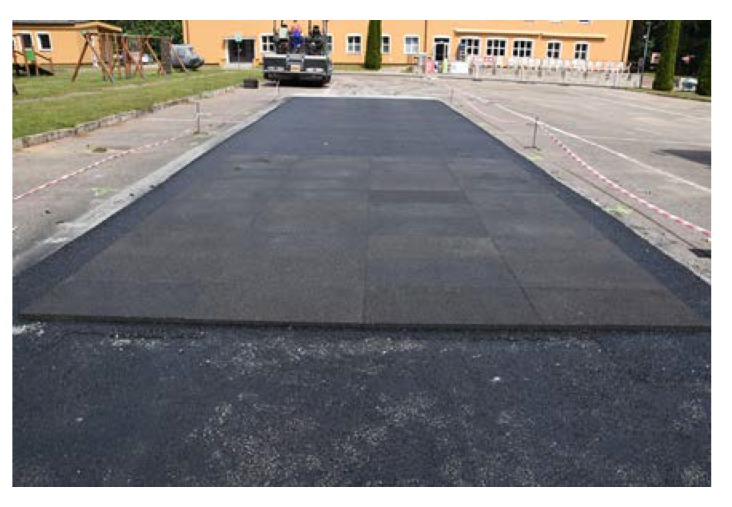
Slabs of Poroelastic Road Surfaces (PERS) polyurethane poroelastic mixture after installation.

Sections composed of SMA, PA and SEPOR mixtures were placed with an asphalt paver and compacted with ordinary road roller. Section with PERS polyurethane poroelastic mixture was constructed by placing prefabricated slabs on the surface of the binder course and bonding them with bitumen emulsion. The dimensions of each section were approximately 8 × 4 m. Selected aspects of the construction phase of test pads for fire tests are presented in Figure 13, Figure 14, Figure 15 and Figure 16.

The main goal of the second test section was (1) to evaluate the performance of the designed poroelastic mixture in real traffic conditions, (2) to evaluate the possible influence of different types of the lower layer on the poroelastic mixture performance and (3) to conduct noise reduction tests at typical traffic speeds. Therefore, the second test section was located on a public road in Gdańsk–Galaktyczna street—which is subjected to constant and significant traffic that includes heavy goods vehicles and buses. In this case, it was decided to place the poroelastic mixture on a single lane of the two-way carriageway, so it was subjected to traffic in one direction. The width of the lane was 3 m. Because of the lack of intersections and exits, no turning movements were occurring. The total length of this test section was 160 m. It was divided into eight subsections with a length of 20 m each that differed in terms of binder content in poroelastic mixture, type of layer underneath and type of bonding technique. All constructed variants are summarized in Table 3.

**Figure 17:**
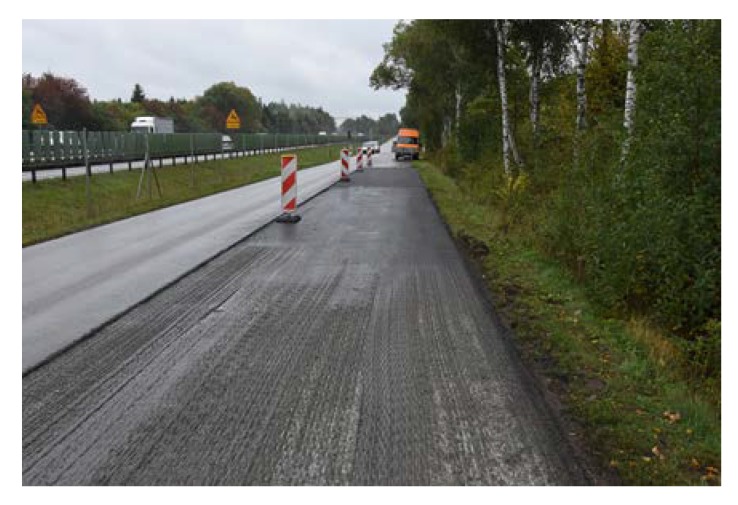
New binder course after surface milling.

**Figure 18:**
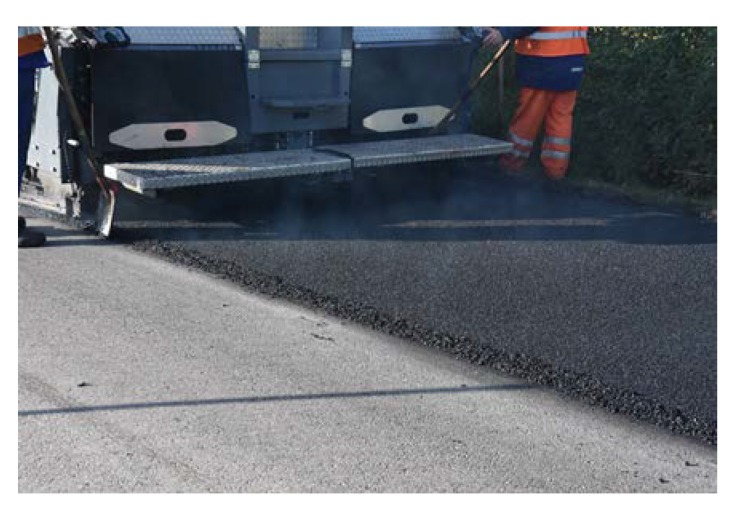
Laying of the poroelastic mixture.

**Figure 19:**
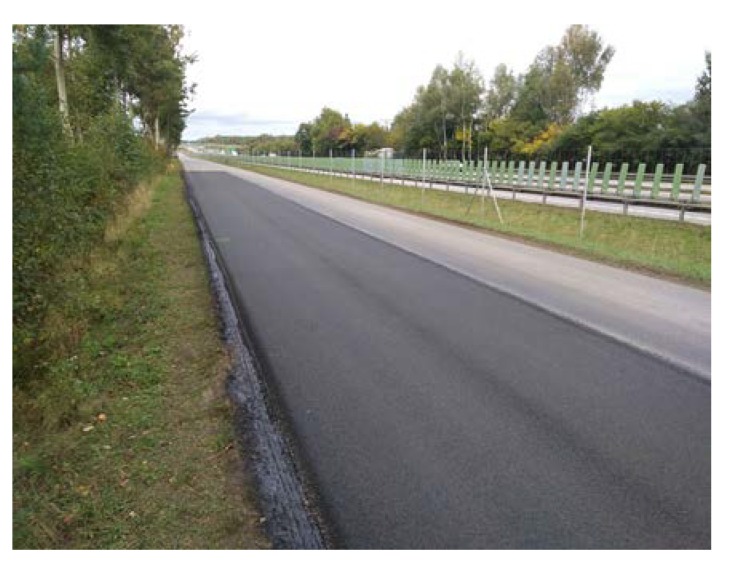
Galaktyczna test section after completion.

**Figure 20:**
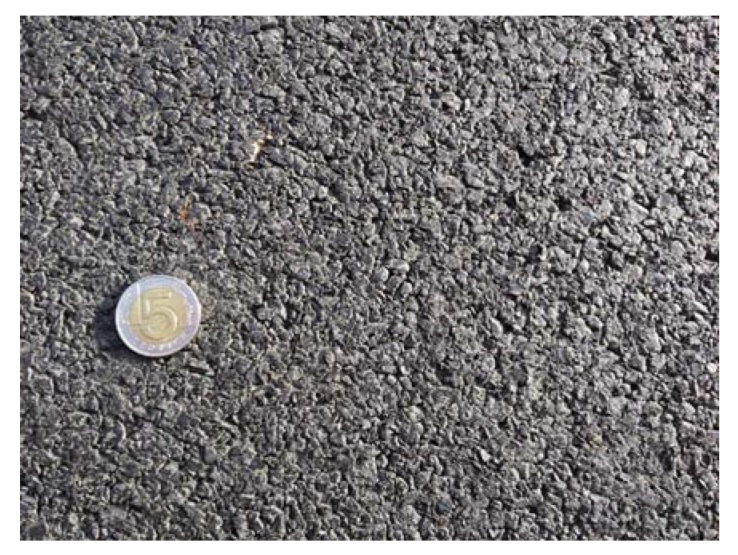
Close-up view of the poroelastic wearing course.

Before placing a new binder course and poroelastic wearing course, the existing pavement was milled out to the depth of 11–12 cm. After that new binder course (made of asphalt concrete AC 16W or stone matrix asphalt SMA 11) was laid and compacted, with additional treatments (surface milling of the new binder course, application of tack coat and glass geogrid) following. Finally, the poroelastic mixture in two variants differing in binder content was placed and compacted. Because the distance between the asphalt plant and the construction site was slightly shorter than previously (approximately 20 km), the time of transport was also shorter and did not exceed 30 min. Selected aspects of the construction phase of the Galaktyczna street trial section are presented in Figure 17, Figure 18, Figure 19 and Figure 20.

**Figure 21:**
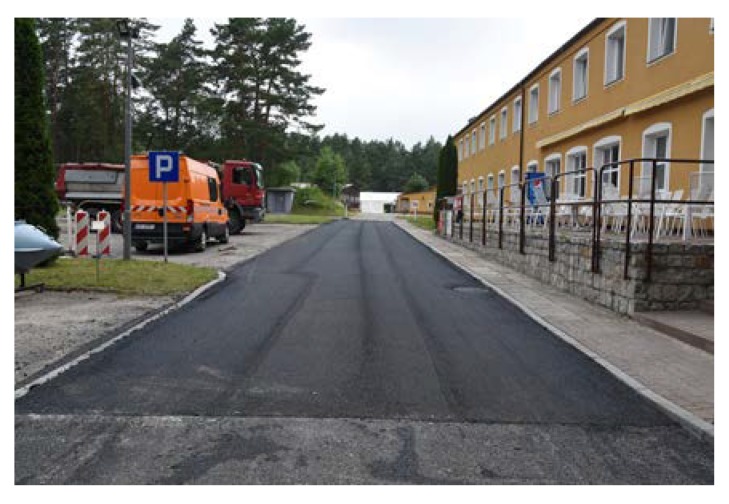
Longitudinal marks caused by paver screed joints on the Dąbrówka trial section.

**Figure 22:**
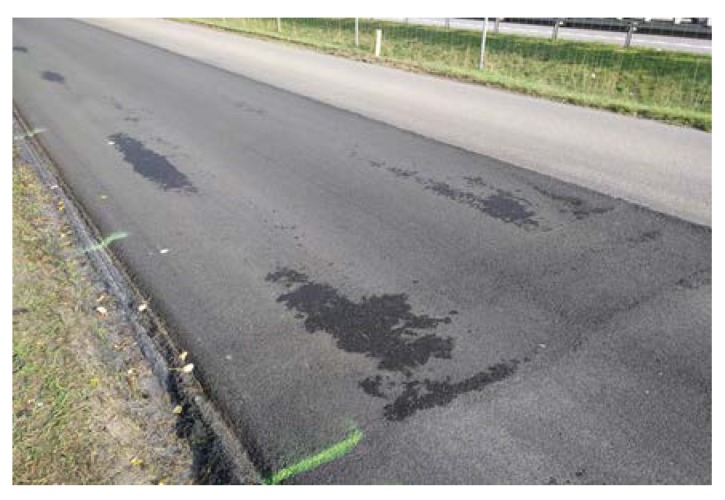
Transvers mark caused by paver stop on the Galaktyczna street trial section.

In summary, during the production of poroelastic mixtures based on highly polymer-modified bitumen, no particular problems occurred. Adding crumb rubber to the pugmill with the use of uncovered conveyor belt was effortless, in spite of the initial fear that lightweight rubber could be blown away by the wind. In all cases, the mixture was uniform and no signs of the binder draining down were observed, even if transport time reached one hour. In general, the texture of the poroelastic layer was quite uniform and without any stains of drained binder (Figure 11 and Figure 15). On the other hand, it should be also noted that the workability and compactibility of the poroelastic mixture were low which resulted in very high sensitivity to paving process disturbances. For example, asphalt paver screed joints induced clearly visible longitudinal marks during construction of the Dąbrówka trial section, which could not be smoothened down with a roller (see Figure 11). Compaction degree in those places was also lower than in other areas. Such defects did not occur when the same paver was used for typical asphalt mixtures (SMA, PA, AC). During construction of the second test section, Galaktyczna, longitudinal marks were avoided by simply laying the poroelastic mixture with paving screed completely folded down. In this case, however, a short stop of the paver during truck change caused visible transverse mark, which again could not be erased with a roller. Moreover, it was also completely impossible to make hand laying or hand corrections of the freshly laid mat (before compaction) without leaving significant marks. Some of the described drawbacks are shown in Figure 21 and Figure 22.

## 4. Field Validation of Poroelastic Pavement Properties

### 4.1. Volumetric and Mechanical Parameters

To verify and compare the characteristics of the material produced in the laboratory and on the asphalt, plant selected laboratory tests were conducted. Volumetric characteristics and inlayer shear strength of the poroelastic layer were compared. Laboratory tests were conducted on two types of specimens:

- Cylindrical specimens with 100 mm diameter, compacted in laboratory (Marshall compactor, 2 × 50 blows), loose mix were collected during laying;
- Cylindrical specimens with 150 mm diameter, drilled out from the compacted layer.

**Figure 23:**
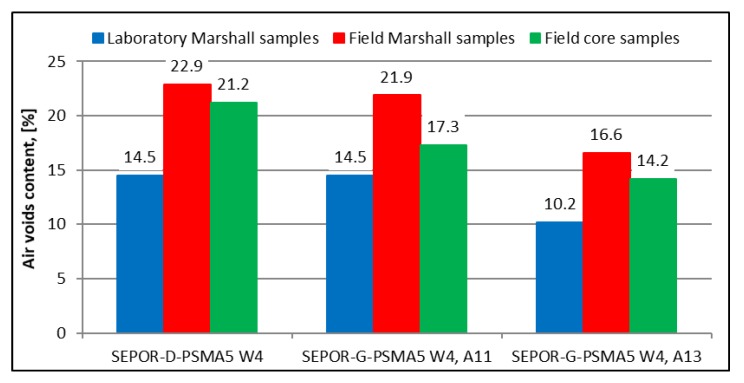
SEPOR-PSMA5 W4 laboratory test results: laboratory and field air voids content comparison.

**Figure 24:**
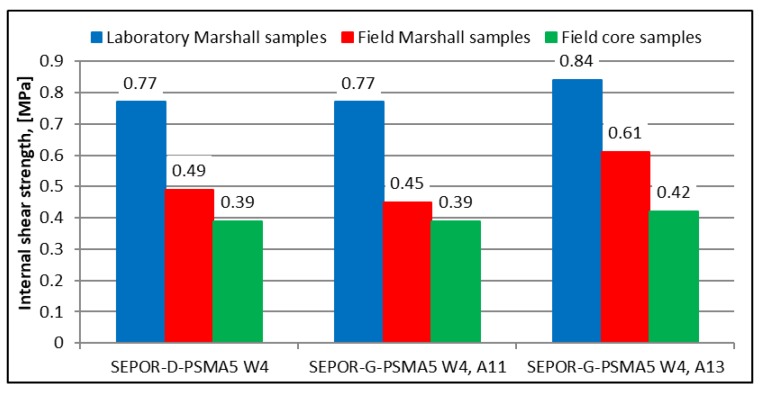
SEPOR-PSMA5 W4 laboratory test results: laboratory and field inlayer shear strength comparison.

Laboratory test results are presented in Figure 23 and Figure 24.

In the case of air voids content, the results (see Figure 23) have shown that the plant mixture production clearly influences the effectiveness of compaction of poroelastic layers. Mixtures produced in the plant are more open (22.9%, 21.9%, 16.6%) compared to mixtures produced in the lab (14.5%, 14.5%, 10.2%) with the same compaction method used. Compaction in the field by typical rollers also produced a more open mixture than compacted in the laboratory even for a mixture produced in the laboratory and in the plant. It means that real production in the plant changes the properties of the poroelastic mixture which should be explained in the future.

Real production of the poroelastic mixture in a plant reduces the inlayer shear strength of the mixture by 30–40% compared to the laboratory production of the poroelastic mixture (see Figure 24). Results from Figure 4 explained the reason for this reduction of the inlayer shear strength as a measure of cohesion of the poroelastic mixture. An increase in air voids content of mixture essentially decreases the inlayer shear strength of the mixture.

### 4.2. Rolling Resistance

Rolling resistance of tires is nowadays receiving more and more attention due to the climatic crisis that humanity faces. It is commonly accepted that lowering the tire rolling resistance by 10% results in a 2–3% reduction of energy consumption and a corresponding reduction of CO_2_ emission by passenger cars. The most spectacular savings are for constant, slow- and medium-speed driving, but the impact of rolling resistance on energy consumption occurs over the entire speed range. Hybrid vehicles benefit from low rolling resistance tires even more, as due to recuperation, the energy losses at variable driving speeds are less, thus rolling resistance dominates also in non-steady state conditions. In the case of electric vehicles, one must remember that, in many countries, electric energy is obtained from fossil fuel power plants and that relatively short operating distance (due to low battery capacity and resistive forces) is the most important issue for those vehicles. So, for all existing road vehicles, the environment benefits from lower rolling resistance.

The rolling resistance of tires is relatively difficult to measure as, for a typical modern tire, the rolling resistance force is less than 1% of tire load. In the case of modern “blue” tires, it may be as low as 0.5–0.6% of the tire load. It is difficult to measure small forces in the presence of large forces that load the measuring system, and in addition, there are many factors interfering with the road measurements such as acceleration (inertia forces), road inclination, wind or temperature changes. On the other hand, it is difficult and often impossible to measure rolling resistance on roadwheel facilities (“drums”) as most road pavements are not suitable for fixing on outer drums and tests performed on smooth steel are not representative.

**Figure 25:**
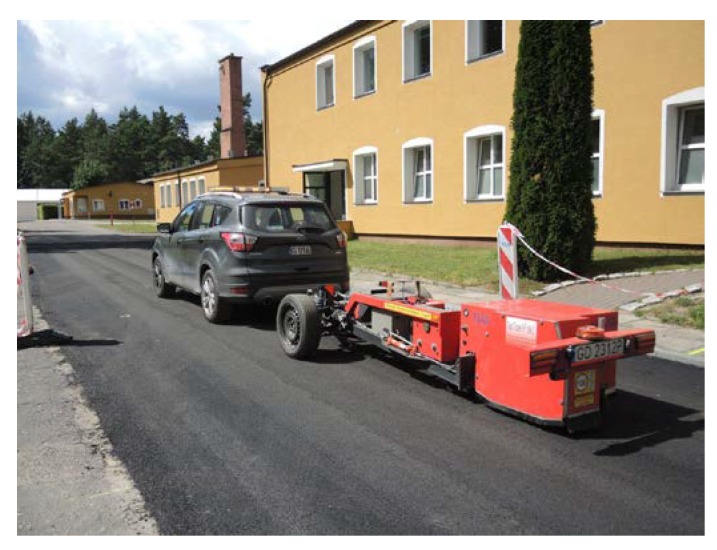
Test trailer R^2^ Mk.2 during measurements of rolling resistance on the Dąbrówka test section.

Gdańsk University of Technology (GUT) designed and built the unique test trailer R^2^ Mk.2 (see Figure 25) that is capable of making road measurements on trafficked roads with very high precision. This trailer, as well as the roadwheel facility, were used to perform rolling resistance measurements reported in this article.

**Figure 26:**
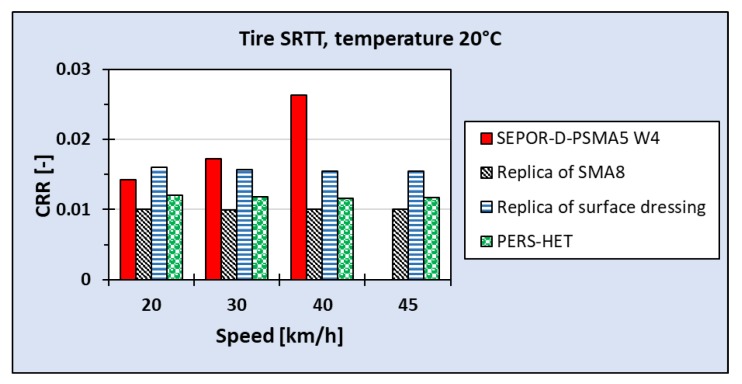
Coefficient of Rolling Resistance (CRR) as measured on the roadwheel facility at 20 °C.

**Figure 27:**
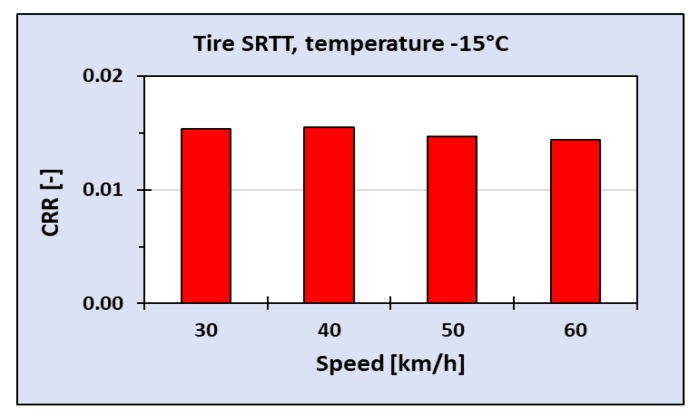
Coefficient of Rolling Resistance (CRR) for SEPOR-D-PSMA5 W4 as measured on the roadwheel facility at −15 °C.

Tests of first samples of SEPOR material were made on the roadwheel facilities in order to estimate rolling resistance before road sections were constructed. As it was mentioned above, the roadwheel facility is not very suitable for testing road pavements due to centrifugal forces that try to tear the pavement from a steel drum. Nevertheless, it was possible to perform tests of SEPOR-D-PSMA5 W4 at speeds up to 40 km/h. Tire SRTT [22] was used as a reference tire. The results are presented in Figure 26. For comparison, three other road pavements were also tested: a replica of a typical SMA8, a replica of very coarse Surface Dressing and a poroelastic road pavement PERS-HET (manufactured in a form of plates by the German company HET, Wiesbaden) that uses a polyurethane binder instead of modified asphalt. It is clearly visible that, for low speeds, the Coefficient of Rolling Resistance (CRR) measured on the SEPOR pavement is very similar to the coefficients measured on other pavements. At higher speeds, the measured CRR for SEPOR rapidly increases and at speed 45 km/h the bulge of the SEPOR pavement due to centrifugal forces was so great that the experiment was stopped due to safety issues. By the opinion of this author, the increase of CRR was not due to the SEPOR properties but due to the deflections of the pavement that was bolted to the drum only at the edges and seriously bulged during tests. This assumption was confirmed by another experiment in which rolling resistance tests were carried out at a very low temperature of −15 °C and the pavement was glued to the drum with bituminous glue. The sample of SEPOR pavement was much stiffer at such a low temperature and bulge did not occur up to the speed of 60 km/h. At this temperature measured rolling resistance has not increased with the speed, just opposite it decreased by a few percent like for other pavements (see Figure 27).

**Figure 28:**
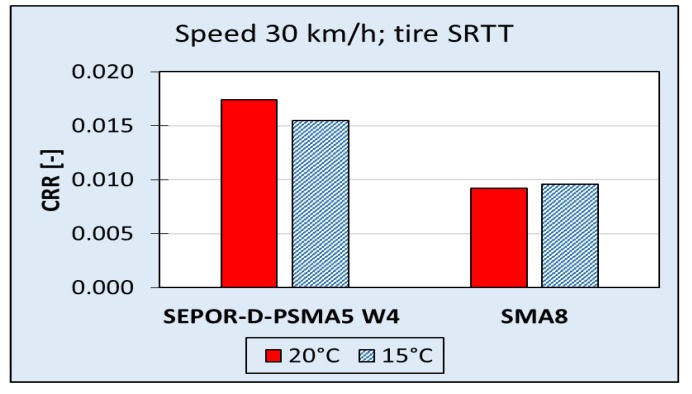
Coefficient of Rolling Resistance (CRR) for SEPOR-D-PSMA5 W4 and reference pavement SMA8 measured at the Dąbrówka section.

Road tests of rolling resistance were performed both on Dąbrówka and Galaktyczna test sections. Despite the fact that the Dąbrówka section was intended mainly for testing SEPOR paving technologies, it was also used to measure rolling resistance at low speeds. Studies have shown that the SEPOR-D-PSMA5 W4 pavement has higher rolling resistance than the reference SMA8 pavement and that this difference is greater at higher temperatures (see Figure 28). The results concerning rolling resistance obtained at the Dąbrówka section were considered to be very disappointing, but it was recognized that perhaps this is due to errors that were made when laying this surface. What should have been emphasized was the task of checking the technology and not checking the product itself.

**Figure 29:**
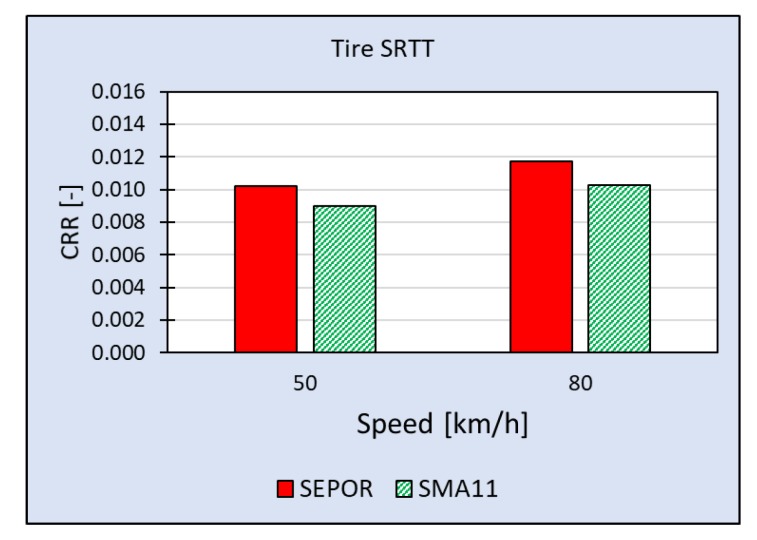
Coefficient of Rolling Resistance for SEPOR-G-PSMA5 W4 and reference pavement SMA11 measured at the Galaktyczna section for tire SRTT at temperature 20 °C.

**Figure 30:**
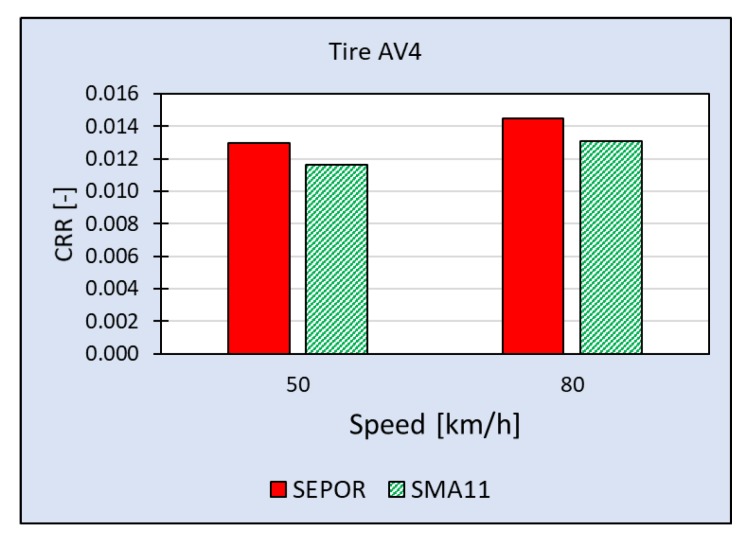
Coefficient of Rolling Resistance for SEPOR-G-PSMA5 W4 and reference pavement SMA11 measured at the Galaktyczna section for tire Avon AV4 at temperature 20 °C.

**Figure 31:**
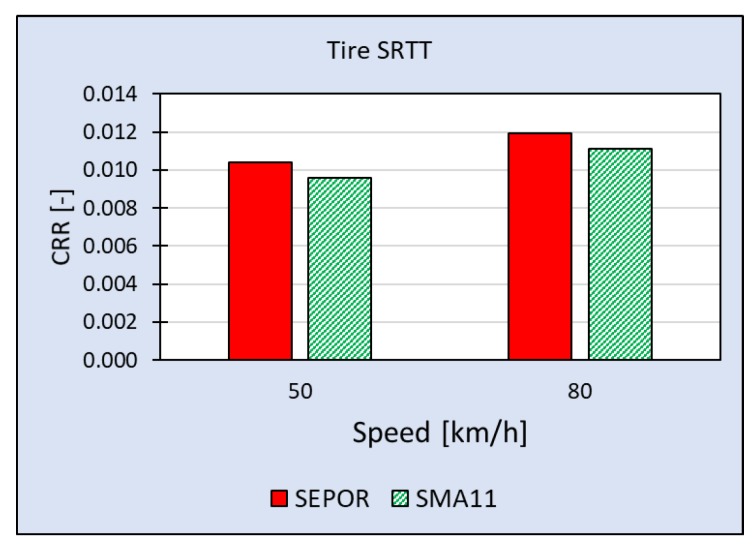
Coefficient of Rolling Resistance for SEPOR-G-PSMA5 W4 and reference pavement SMA11 measured at the Galaktyczna section for tire SRTT at temperature 10 °C but normalized to 25 °C.

The most comprehensive measuring campaign so far was executed on SEPOR-G-PSMA5 W4 at the Galaktyczna test section. This section is placed on a straight, flat road and it is possible to perform tests with speed up to 80 km/h. What is also important this test section is trafficked and it is possible to evaluate its wear. The first round of tests was performed when the pavement was one week old and the second round was performed five weeks later. Results of rolling resistance measurements for two reference tires [23] obtained during the first round of measurements when the air temperature was 20 °C are presented in Figure 29 and Figure 30. The second round of measurements was performed at a lower temperature (10 °C) and only for one tire—SRTT. The results are presented in Figure 31.

**Figure 32:**
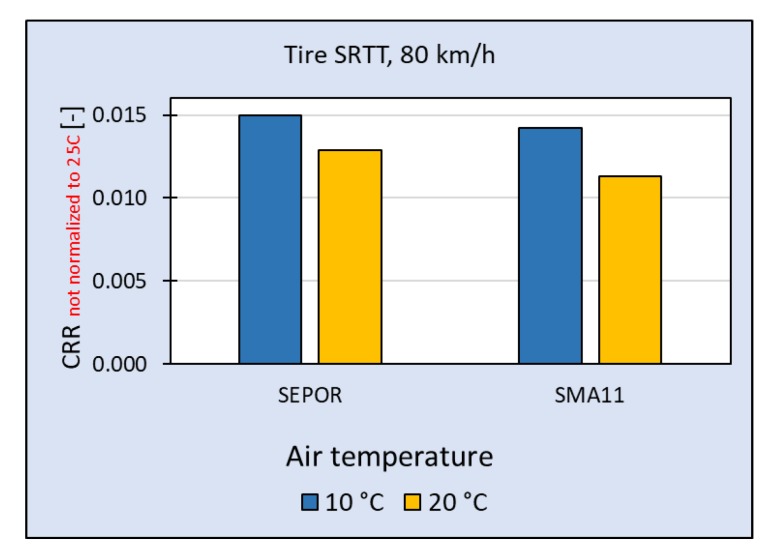
Coefficient of Rolling Resistance for SEPOR-G-PSMA5 W4 and reference pavement SMA11 measured at the Galaktyczna section for tire Avon AV4; values not normalized to 25 °C.

In Figure 32, the results of rolling resistance measurements at temperature 10 °C and 20 °C are compared, but unlike in other figures, CRR is not normalized to temperature 25 °C. Although rolling resistance on SEPOR-G-PSMA5 W4 pavement is higher than on reference pavement SMA11, the difference is much less than on Dąbrówka test section. The measurements indicate also that rolling resistance on the SEPOR pavement is not increasing with the decrease of temperature as much as in the case of SMA11.

All investigations performed so far indicate, that rolling resistance on SEPOR type pavement is higher than on conventional pavements like SMA8 or SMA11, but when the paving process is well done, the difference is not that big. All measurements indicate also, that the increase of rolling resistance at low temperatures is less for SEPOR than for other pavements. The increase of rolling resistance must be attributed to energy losses that are developed (probably mostly in the binder) when rubber aggregate in the SEPOR material is compressed by the tire. The rubber is highly elastic, but the binder is rather plastic, which means that there is a large hysteresis in the mixture.

### 4.3. Acoustical Properties

The main component of road traffic noise is tire/road noise, which depends on the design parameters of the tires, the pavements and the traffic conditions. In the second half of the 20th century, great progress was made in silencing engine and transmission noise, which meant that these sources, traditionally the loudest, have become less important than the noise generated during tire-road interaction. Despite extensive development work carried out in the tire industry, it should not be expected that further significant noise reduction by upgrading conventional tires will be possible. The optimization of the tread pattern is already very advanced and other tire parameters that affect noise (rubber compound hardness, tire dimensions, belt construction) are optimized due to other equally important criteria (safety, durability, fuel economy). It seems, therefore, that currently the greatest attention should be directed to the development of “low noise” road surfaces.

Tire/road noise generation mechanisms can be classified into two main groups [1]. One group is directly related to mechanical vibrations of the tire and is structure-borne (including the tread impact and adhesion mechanisms). The other group is related to airflow phenomena and is air-borne. It must be stressed that for traffic noise impact considerations, not only generation mechanisms, but also the propagation of the sound from the source to the recipient is of great importance. Porous pavements help to damp acoustical energy on the propagation path.

To put it simply, it can be stated that vibration-related mechanisms of noise generation are dependent on the shape, stiffness and damping that characterize the elements of the tire (tread, sidewalls, carcass) and the road pavement. In the case of aerodynamic mechanisms, on the other hand, the porosity of the pavement, the shape of the tread groove system and the texture of the pavement are of great importance.

The idea of poroelastic pavements is based on the principle that at the same time the surface has considerable elasticity, which reduces the impact mechanisms of noise generation and high drainage properties (open pores), which reduces aerodynamic mechanisms. Poroelastic pavements based on a polyurethane binder were found to be extremely quiet [24,25,26].

**Figure 33:**
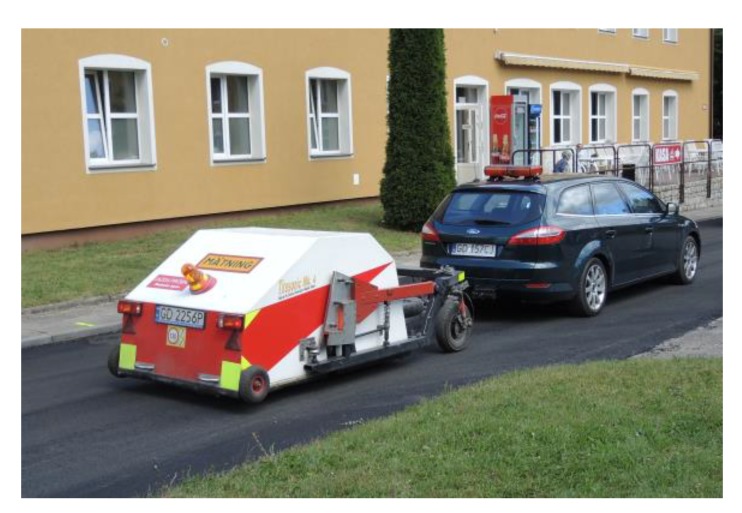
Test trailer Tiresonic Mk.4.

**Figure 34:**
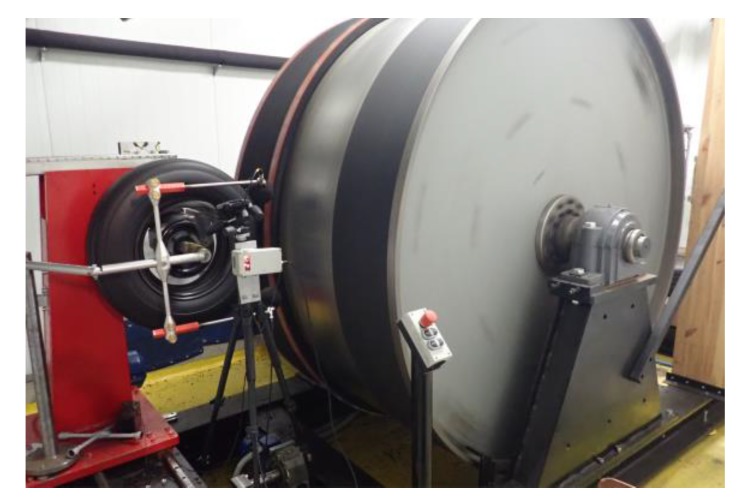
Roadwheel facility at GUT.

**Figure 35:**
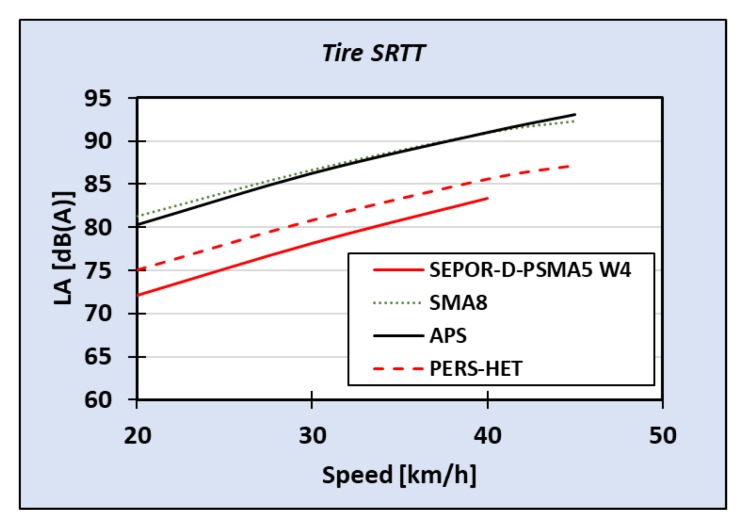
Results of laboratory tire/road noise measurements for tire SRTT.

**Figure 36:**
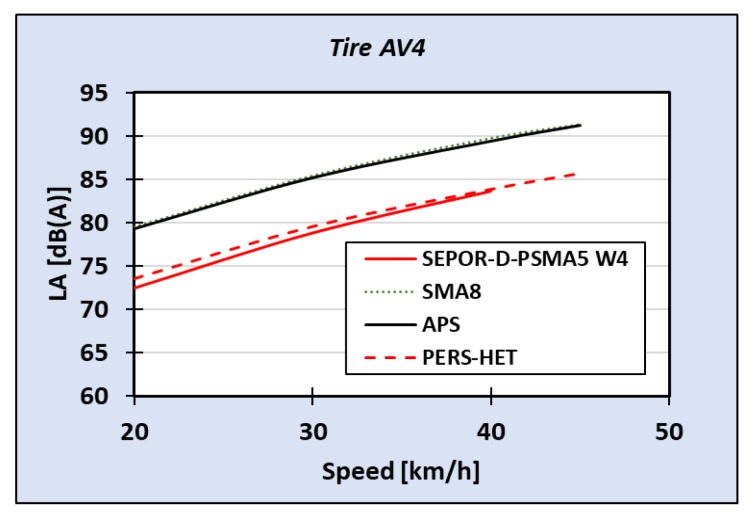
Results of laboratory tire/road noise measurements for tire AV4.

Pavements of SEPOR type were tested by GUT both on the road using test trailer Tiresonic Mk.4 (see Figure 33) and in the laboratory on roadwheel facility (see Figure 34). Results of laboratory tire/road noise tests indicated that noise reduction obtained on SEPOR type pavement is similar to on PERS-HET pavement that was developed in the PERSUADE project (Seventh Framework Program, project *Poroelastic Road Surface: an innovation to Avoid Damages to the Environment*) on the base of polyurethane binder. Unfortunately, noise reduction in comparison to conventional pavements like SMA 8 and Surface Dressing was lower than expected, at about 8 dB (see Figure 35 and Figure 36).

Measurements performed on road test sections with the Tiresonic Mk.4 trailer confirmed the laboratory results. At the Dąbrówka test section, reduction of tire/road noise was 9.2 dB for speed 30 km/h and 8.4 dB for speed 50 km/h. On the Galaktyczna test section, reduction was smaller, at 5.6 dB for tire SRTT and 4.4 dB for tire Avon AV4 [27].

### 4.4. Impact on the Spread of Fuel Spills Fires

Poroelastic road pavements contain a considerable amount of rubber that is flammable so, in theory, they may promote the spread of spilled fuel fires during car accidents. However, already 20 years ago Meiarashi [28], discovered that this is not a case with poroelastic road pavements that use polyurethane as a binder. A few years ago, within the PERSUADE project, it was also found that contrary to the expectations of a few experts, fuel fires on poroelastic pavements do not lead to excessive emission of hydrogen cyanide (HCN) [4].

Despite the fact that both the Meiarashi experiment and experiments within the PERSUADE project showed that poroelastic pavement hampers the spread of fire, it was decided to carry out tests also on SEPOR surfaces. The reason was to check whether replacing the polyurethane binder with modified asphalt would not increase the spread of fire. Experiments were performed at ex-military grounds at the Dąbrówka site. Four different road pavements were tested in exactly the same way. There were: SEPOR-D-PSMA5 W4, PERS-HET, Stone Mastic Asphalt SMA 8 and Drainage Pavement without rubber aggregate.

**Figure 37:**
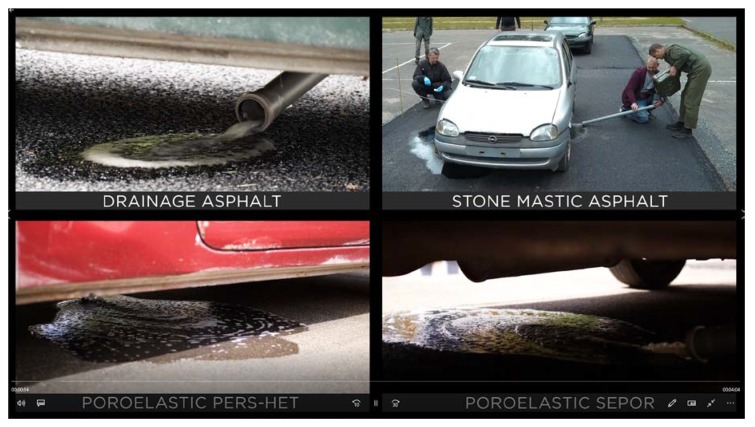
Fuel spills under test cars.

Four very similar Opel Corsa cars were used during the tests. Each car was placed on a 4 m × 8 m test field paved with one of the above-mentioned surfaces and 20 L of gasoline were spilled under it. Gasoline was ignited 30 s after the spill with 25 g napalm charges. Tests were performed with intervals of 30 min so weather conditions were very similar. All experiments were recorded by cameras. In Figure 37, fuel spills are presented. It is clearly visible that on poroelastic and drainage pavements fuel, penetrates the surface so the “fuel lake” is very small. On dense pavement, as SMA8, the fuel cannot penetrate the pavement so a spot of spilled fuel surrounds the car.

**Figure 38:**
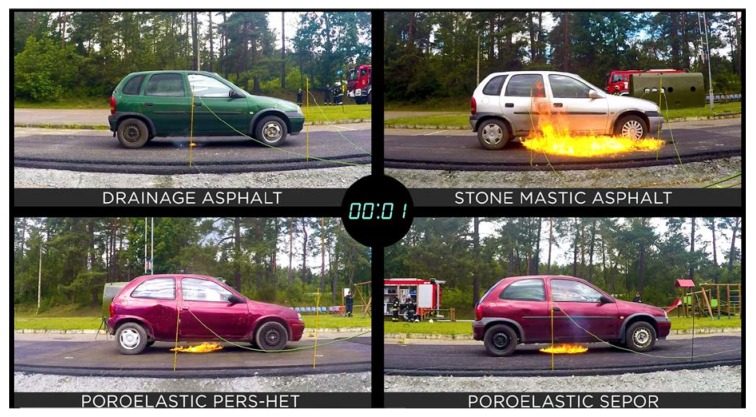
Development of fire 1 s after ignition.

**Figure 39:**
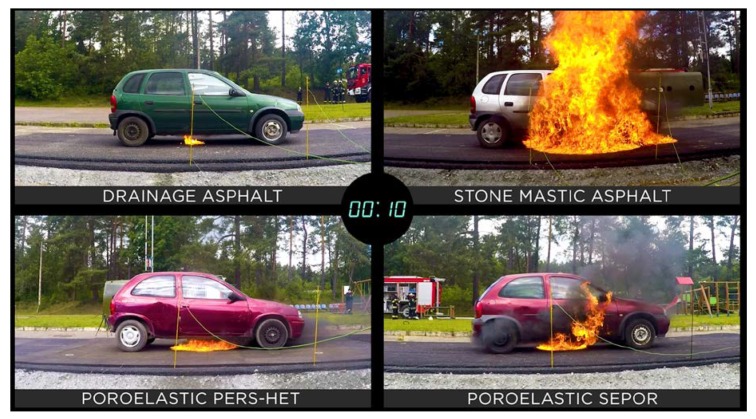
Development of fire 10 s after ignition.

**Figure 40:**
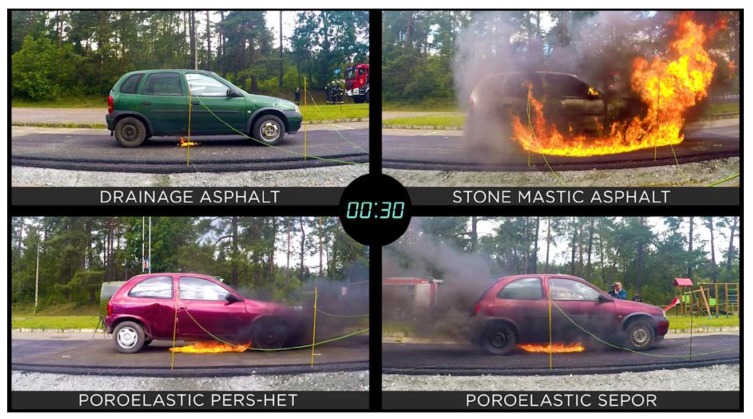
Development of fire 30 s after ignition.

In Figure 38, Figure 39 and Figure 40, the development of fire is presented. One second after ignition flame under the car standing on drainage asphalt was very small, flames under cars standing on poroelastic pavements were somewhat higher but also not dangerous, while in the case of SMA 8 it would already be dangerous to leave the car. Ten seconds after ignition car standing on SMA 8 was already burning with flames as high as 2–3 m making the evacuation of passengers nearly impossible. Unlike SMA 8 it would still be possible to leave cars standing on poroelastic pavements, and the flame under the car standing on drainage asphalt was very small and not dangerous. A similar situation was recorded 30 s after the ignition.

Summarizing the results, a spill of 20 L gasoline on drainage asphalt burning was not able to ignite the car so after 10 min the experiment was stopped. On poroelastic pavements, it would be possible to leave the car during the first 2 min of fire. After 2, min the interiors of cars started to burn due to heat coming through the floors. In the case of typical dense pavement like SMA8, after only 3 s, the fire cut off the possibility of evacuation through the door, and after 30 s, the interior of the vehicle was on fire.

The above experiment shows that drainage pavements have very favorable fire properties and may be used in places where fire risk is very high (tunnels, fuel stations, etc). Poroelastic pavements like SEPOR exhibit also very good fire properties despite the high contents of rubber. Regardless of whether modified asphalt or polyurethane is used as the binder, their fire properties are similar.

## 5. Conclusions

On the basis of the conducted laboratory research, initial field trials and validation of the poroelastic pavement made with crumb rubber, mineral aggregate and highly polymer-modified bitumen, the following conclusions can be formulated:

(1) Highly polymer-modified asphalt binders (HiMA 45/80-80) are promising in terms of obtaining reliable and durable poroelastic mixtures and can be used as a replacement of the previously used polyurethane binders, which are expensive and difficult to use.
(2) The adopted test procedure for poroelastic mixtures, which involved testing of inlayer shear strength, enabled the design of mixtures with a proper level of internal cohesion and, simultaneously, acceptable poroelastic characteristics.
(3) Field trials that involved ordinary asphalt batch plants have shown that production of the poroelastic mixture with the addition of 15% of crumb rubber using installation normally used for reclaimed asphalt pavement is possible.
(4) Laying and compacting of the poroelastic mixture with ordinary paving machines and rollers was not complicated and did not require any modifications in the equipment, but compaction methods must be revised to obtain better consistency of field compaction levels with laboratory results.
(5) Poroelastic mixtures produced in the plant are more open (have a higher air voids content) compared to poroelastic mixtures produced in the laboratory, even if the same compaction method was used; Marshal hammer in the laboratory.
(6) Poroelastic mixtures produced in the plant characterized 30–40% lower cohesion measured by the Leutner apparatus as an inlayer shear strength compared to the mixture produced in the laboratory.
(7) Poroelastic pavements of the SEPOR type tested so far exhibit lower noise reduction 6–9 dB instead of the desired 10–12 dB, so further changes to the mixture and paving technology should be introduced.
(8) Rolling resistance of passenger car tires on SEPOR type pavements is at about 10% higher than on reference road pavement SMA8.
(9) SEPOR type pavement exhibit very promising properties in case of spill fuel fires hampering the spread of fire and giving a considerable time margin for the evacuation of passengers.

## Figures and Tables

**Table 1:** Properties of SBS-highly modified bitumen.

Property		45/80-80
Penetration at 25 °C, 0.1 mm, acc. to PN-EN 1426	Original	53
	RTFO	40
R&B Temperature, [°C], acc. to PN-EN 1427	Original	78.7
	RTFO	87.8
Performance Grade, acc. to AASHTO M 320		82-22
Resistance to heavy traffic load, acc. to AASHTO M 332		E

**Table 2:** SEPOR-PSMA5 W4 poroelastic mixture composition.

Mix Ingredient	Rubber–Mineral Aggregate Mix (%)	Asphalt–Rubber–Mineral Aggregate Mix (%)			
Coarse aggregate 2/5	72	65.2	63.8	62.4	60.9
Fine aggregate 0/2	6	5.4	5.3	5.2	5.1
Limestone filler	7	6.3	6.2	6.1	5.9
Crumb rubber 1/4	10	9.1	8.9	8.7	8.5
Crumb rubber 0.5/2	5	4.5	4.4	4.3	4.2
Cellulose fibers	-	0.4	0.4	0.4	0.4
45/80-80 binder	-	9.0	11.0	13.0	15.0

**Table 3:** Summary of construction variants for the Galaktyczna trial section.

Subsection Designation	1	2	3	4	5	6	7	8
Amount of binder in poroelastic mixture, % m/m	11				13			
Application of glass geogrid	No	No	No	Yes	Yes	No	No	No
Amount of tack coat, kg of bitumen residue per 1 m^2^	0.2		0.3				0.2	
Type of tack coat	Bitumen emulsion C60 BP3 ZM							
Application of longitudinal milling	No	No	Yes	Yes	Yes	Yes	No	No
Type of lower layer	AC16W 35/50	SMA 11 45/80-55						AC16W 35/50
